# Correction to: Location of studies and evidence of effects of herbivory on Arctic vegetation: a systematic map

**DOI:** 10.1186/s13750-022-00265-z

**Published:** 2022-06-03

**Authors:** Eeva M. Soininen, Isabel C. Barrio, Ragnhild Bjørkås, Katrín Björnsdóttir, Dorothee Ehrich, Kelly Hopping, Elina Kaarlejärvi, Anders Lorentzen Kolstad, Svetlana Abdulmanova, Robert G. Björk, C. Guillermo Bueno, Isabell Eischeid, Rebecca Finger Higgens, Jennifer Sorensen Forbey, Charles Gignac, Olivier Gilg, Michael den Herder, Hildur Søndergaard Holm, Bernice C. Hwang, Jane Uhd Jepsen, Stefaniya Kamenova, Ilona Kater, Amanda M. Koltz, Jeppe Aagaard Kristensen, Chelsea J. Little, Petr Macek, Karen Marie Mathisen, Daniel Metcalfe, Jesper Bruun Mosbacher, Martin Alfons Mörsdorf, Taejin Park, Jeffrey Propster, Aradhana Roberts, Emmanuel Serrano Ferron, Marcus P. Spiegel, Mariana Tamayo, Maria W. Tuomi, Megha Verma, Katariina Elsa Maria Vuorinen, Maria Väisänen, René Van der Wal, Megan Wilcots, Nigel Yoccoz, James D. M. Speed

**Affiliations:** 1 Department of Arctic and Marine Biology, UiT-The Arctic University of Norway, 9037 Tromsø, Norway; 2 Faculty of Environmental and Forest Sciences, Agricultural University of Iceland, Keldnaholt, 112 Reykjavík, Iceland; 3 Centre for Biodiversity Dynamics, Department of Biology, Norwegian University of Science and Technology, 7491 Trondheim, Norway; 4 Department of Biological and Environmental Sciences, University of Gothenburg, PO Box 461, 405 30 Gothenburg, Sweden; 5 Human-Environment Systems, Boise State University, Boise, ID 83725 USA; 6 Organismal and Evolutionary Research Programme, University of Helsinki, 00014 Helsinki, Finland; 7 Department of Natural History, NTNU University Museum, Norwegian University of Science and Technology, 7491 Trondheim, Norway; 8 Dynamics of Arctic Ecosystems Lab, Arctic Research Station, Institute of Plant and Animal Ecology Ural Branch of Russian Academy of Sciences, Zelenaya Gorka Str., 21, Labytnangi, 629400 Russia; 9 Department of Earth Sciences, University of Gothenburg, 405 30 Gothenburg, Sweden; 10 Gothenburg Global Biodiversity Centre, 405 30 Gothenburg, Sweden; 11 Department of Botany, Institute of Ecology and Earth Sciences, University of Tartu, 51005 Tartu, Estonia; 12 Norwegian Polar Institute, Fram Centre, 9296 Tromsø, Norway; 13 Ecology, Evolution, Ecosystems and Society, Dartmouth College, Hanover, NH 03755 USA; 14 Department of Biological Sciences, Boise State University, Boise, ID 83725 USA; 15 Centre d’études Nordiques and Plant Science Department, Université Laval, 2425 rue de l’Agriculture, Québec City, Québec Canada; 16 UMR 6249 Chrono-Environnement, Université de Bourgogne Franche-Comté, 25000 Besançon, France; 17 Groupe de Recherche en Ecologie Arctique, 21440 Francheville, France; 18 European Forest Institute, Yliopistokatu 6 B, 80100 Joensuu, Finland; 19 Department of Physical Geography and Ecosystem Science, Lund University, Sölvegaten 12, 22362 Lund, Sweden; 20 Department of Arctic Ecology, Norwegian Institute for Nature Research, Fram Centre, 9297 Tromsø, Norway; 21 Centre for Ecological and Evolutionary Synthesis, University of Oslo, Oslo, Norway; 22 Department of Biosciences, University of Durham, Stockton Road, Durham, DH1 3LE UK; 23 Department of Biology, Washington University in St. Louis, St. Louis, MO 63130 USA; 24 The Arctic Institute, Washington, DC 20009 USA; 25 School of Geography and the Environment, University of Oxford, Oxford, OX1 3QY UK; 26 School of Environmental Science, Simon Fraser University, Burnaby, BC V5A 1S6 Canada; 27 Institute of Hydrobiology, Biology Centre of Czech Academy of Sciences, Na Sadkach 7, 370 05 Ceske Budejovice, Czech Republic; 28 Faculty of Science, University of South Bohemia, Branisovska 1760, 37005 Ceske Budejovice, Czech Republic; 29 Department of Forestry and Wildlife Management, Inland Norway University of Applied Sciences, 2418 Elverum, Norway; 30 Department of Ecology and Environmental Science, Umeå University, Linnaeus väg 6, 901 87 Umeå, Sweden; 31 Department of Biology, University of Freiburg, 79104 Freiburg, Germany; 32 NASA Ames Research Center, Moffett Field, CA 94035 USA; 33 Bay Area Environmental Research Institute, Moffett Field, CA 94035 USA; 34 Center for Ecosystem Science and Society, Northern Arizona University, Flagstaff, AZ 86011 USA; 35 Department of Biological Sciences, Northern Arizona University, Flagstaff, AZ 86011 USA; 36 Wildlife Ecology and Health Group (WE&H) and Servei d’Ecopatologia de Fauna Salvatge (SEFaS), Departament de Medicina I Cirurgia Animals, Universitat Autònoma de Barcelona, Bellaterra, 08193 Barcelona, Spain; 37 Environment and Natural Resources, University of Iceland, Sturlugata 7, 101, Reykjavík, Iceland; 38 Institute of Zoology, Zoological Society of London, Regent’s Park, London, NW1 4RY UK; 39 Ecology and Genetics Research Unit, University of Oulu, P. O. Box 3000, 90014 Oulu, Finland; 40 Arctic Centre, University of Lapland, P. O. Box 122, 96101 Rovaniemi, Finland; 41 Department of Ecology, Swedish University of Agricultural Sciences (SLU), Ulls väg 16, 75651 Uppsala, Sweden; 42 Department of Ecology, Evolution, and Behavior, University of Minnesota, 140 Gortner Laboratory, 1479 Gortner Ave., St. Paul, MN 55108 USA

## Correction to: Environ Evid (2021) 10:25 https://doi.org/10.1186/s13750-021-00240-0

Following publication of the original article [1], the authors reported that full author names need to appear in the article. Hence, we have update full author name in this correction. The original article [1] has been corrected.
